# Manual Therapy and Exercise Have Similar Outcomes to Corticosteroid Injections in the Management of Patients With Subacromial Pain Syndrome: A Systematic Review and Meta-Analysis

**DOI:** 10.7759/cureus.48907

**Published:** 2023-11-16

**Authors:** Stefanos Karanasios, Georgios Baglatzis, Ioannis Lignos, Evdokia Billis

**Affiliations:** 1 Department of Physiotherapy, University of West Attica, Athens, GRC; 2 Department of Physiotherapy, University of Patras, Patras, GRC

**Keywords:** systematic review and meta analysis, physiotherapy, pain, function, shoulder impingement syndrome

## Abstract

Subacromial pain syndrome (SAPS) is the most frequent diagnosis in patients with shoulder pain presenting with persistent pain and significant functional decline. Although exercise and manual therapy (EMT) and corticosteroid injections provide first-line treatment options, evidence for the best management of SAPS remains inconclusive. We aimed to evaluate the effectiveness of EMT compared with corticosteroid injections on disability, recovery rates, and pain in patients with SAPS through a systematic review and meta-analysis approach.

PubMed, Cumulative Index to Nursing and Allied Health Literature (CINAHL), Physiotherapy Evidence Database (PEDro), ScienceDirect, the Cochrane Library, and grey literature databases were searched. Only randomized controlled trials evaluating the effectiveness of EMT alone or as an additive intervention compared to corticosteroid injections were included. Methodological quality was evaluated with the PEDro score and certainty of evidence with the Grading of Recommendations Assessment, Development, and Evaluation (GRADE) approach.

In total, 8 trials with 946 patients were included. EMT presented no difference in disability compared with corticosteroid injections at very short- (standardized mean difference {SMD}: 0.19; 95%CI: -0.20, 0.58), short- (SMD: -0.16; 95%CI: -0.58, 0.25), mid- (SMD: -0.14; 95%CI: -0.44, 0.16), and long-term (SMD: 0.00; 95%CI: -0.25, 0.25) follow-up. No difference was found between the comparators in self-perceived recovery at very short- (risk ratio: 0.93; 95%CI: 0.71, 1.21) and mid- (risk ratio: 0.98; 95%CI: 0.90, 1.07) follow-up and in pain rating at very short- (SMD: -0.18; 95%CI: -0.73, 0.38), short- (SMD: 0.05; 95%CI: -0.26, 0.37), and long-term (SMD: 0.04; 95%CI: -0.26, 0.34) follow-ups. The addition of corticosteroid injections to EMT provided no better results in shoulder disability compared with EMT (SMD: 0.45; 95%CI: -0.47, 1.37) or corticosteroid injections alone (MD: 2.70; 95%CI: -7.70, 13.10) in the mid-term.

Based on very low to moderate certainty of evidence, EMT has similar effects to corticosteroid injections on improving all outcomes in patients with SAPS at all follow-up periods. Based on low certainty of evidence the combination of both interventions does not change the treatment outcome compared with each intervention alone.

## Introduction and background

Subacromial pain syndrome (SAPS), traditionally known as shoulder impingement syndrome, is the most commonly reported diagnosis of shoulder pain, accounting for 11 to 12 per 1000 general practice consultations [1,2]. The lifetime prevalence of SAPS ranges between 7% and 26% in the general population and increases with age [3]. Several structures are thought to be involved in the pathogenesis of SAPS, such as the acromion, rotator cuff tendons, acromioclavicular joint, glenohumeral ligaments, and capsule [2,4]. Hence, a range of pathological conditions are considered part of the SAPS, including bursitis, rotator cuff tendinopathy with or without partial tear, calcific tendinitis, biceps tendinitis, tendon degeneration, etc. [5,6]. The clinical presentation of SAPS may include pain, decreased shoulder movement and function usually experienced during shoulder elevation, and external rotation [2]. It has been associated with persistent symptoms even two years after the onset of the condition, causing significant disability and economic impact [3,7].

Current published guidelines provide inconsistent recommendations for the best management of patients with SAPS [2,7,8]. Conservative approaches are considered first-line treatments, presenting similar results to surgical interventions [2,9]. The most commonly used nonsurgical interventions include a multimodal physiotherapy programme with exercise and manual therapy (EMT) or corticosteroid injections [10,11]. Several studies have shown that EMT can significantly improve pain and function in patients with SAPS [2,12,13]; however, the extent of their effectiveness remains unclear [2,8,13]. Similarly, despite corticosteroid injections being widely used to reduce inflammation resulting from SAPS, their efficacy is debated [14]. Older reviews highlighted that corticosteroid injections provide only short-term benefits in reducing pain intensity compared with physiotherapy or placebo, while there are no clear benefits in favour of corticosteroid injections at the mid- and long-term follow-up times [8,10,15]. It seems that an updated systematic review of the effectiveness of EMT compared with corticosteroid injections is necessary to facilitate clinical decisions in prescribing the best treatment options for patients with SAPS.

The purpose of the present review was to evaluate the effectiveness of a multimodal treatment approach, including EMT compared with corticosteroid injections in the management of SAPS in the very short-term (≤2 months), short-term (>2 months to ≤3 months), mid-term (>3 to <12 months) and long-term (⩾12 months) follow-up times [16]. We also aimed to synthesise the evidence regarding interventions (type, mode, frequency, etc.) and followed an established approach for assessing the certainty of evidence [16].

## Review

Methodology

We followed the Preferred Reporting Items for Systematic Reviews and Meta-Analyses (PRISMA) guidelines in the conduction and reporting of the present review. The study protocol was registered in the Prospective Register of Systematic Reviews (PROSPERO) with registration number CRD42023471214.

Study Design

We included only randomised controlled trials (RCTs) assessing the effectiveness of EMT used alone or in combination with other interventions in patients with SAPS. We did not use any language restrictions.

Participants

Eligible participants considered were patients over 18 years old, both men and women and with clinical symptoms of SAPS. The diagnostic labels considered as part of the condition included rotator cuff tendinopathy, painful arc syndrome, subacromial bursitis, shoulder impingement, rotator cuff tendinosis or tendinitis, and contractile dysfunction [2,7]. We excluded patients treated surgically or with frozen shoulder, posttraumatic shoulder pain, posttraumatic rotator cuff tear, shoulder instability, shoulder osteoarthritis, rheumatoid diseases, neck pain, cancer, neuropathic pain, and neurological conditions.

Interventions

We included studies using any type of manual therapy (joint mobilisation, manipulation, soft tissue mobilisation, or massage) in combination with any type of exercise (stretching, range of motion, stabilisation, strengthening, proprioceptive, postural, motor control, or a combination of these) used alone or as an additive intervention in adult patients with SAPS.

Comparisons

Studies using comparisons of corticosteroid injections (guided or unguided) used alone or in combination with other conservative interventions were included.

Outcomes

The primary outcomes included (i) pain intensity using the Visual Analogue Scale (VAS) score or Numeric Pain Rating Scale (NPRS); (ii) disability using the Disability of the Arm, Shoulder, and Hand (DASH) questionnaire, the Western Ontario Disability Index (WORC), the Oxford Shoulder Scale (OSS), the Shoulder Pain and Disability Index (SPADI), or Constant-Murley Score; (iii) global assessment of treatment success; and (iv) adverse events. Secondary outcome measures included (i) range of motion (⁰) and (ii) quality of life (QoL).

Search Strategy

We searched PubMed, Cumulative Index to Nursing and Allied Health Literature (CINAHL), Physiotherapy Evidence Database (PEDro), ScienceDirect, the Cochrane Library, grey literature databases, and clinical trial registries from inception to September 2023 (see Appendices).

Data Selection

After searching the databases, the results were imported into EndNote version X9, and two researchers (GM and SK) thoroughly reviewed titles, abstracts, and full texts against the eligibility criteria in two stages [17]. The researchers independently selected eligible studies and subsequently cross-checked their findings. In case of any disagreement, a third reviewer (IL) was consulted, and any discrepancies were resolved through a consensus process.

Data Extraction

The two researchers (GM and SK) independently conducted the data extraction using a standardised data extraction form including details about the authors, publication year, sample size, interventions, comparative interventions, outcomes, follow-ups, and results. Any discrepancy was resolved through a consensus process with a third reviewer (IL).

Risk of Bias

Two independent reviewers (EB and IL) evaluated the methodological quality of the eligible studies using PEDro criteria. Each study was rated with a score from 0 to 10, based on the number of criteria satisfied [18,19]. The methodological quality of each trial was considered ‘poor’ for scores ⩽4, ‘moderate’ for scores 5 or 6, and ‘high’ for scores ⩾7 [18,20]. Any difference was resolved using a consensus approach with the help of a third reviewer (SK).

Data Analysis, Synthesis, and Summary of Findings

The meta-analyses were estimated using RevMan version 5.4, a software developed by the Cochrane Collaboration (London, UK). Outcome data for pain intensity and disability were transformed to 0-100-point scales, calculating mean differences (MD) or standardized mean differences (SMD) with 95% confidence intervals (CIs) where necessary as measures of the treatment effect. Accordingly, risk ratios with 95% CIs were used for dichotomous data. Considering that increased clinical and methodological heterogeneity was found among interventions, a random-effects model was used to pool the studies’ outcomes. For the meta-analyses, the RevMan version 9 was used.

To evaluate the risk of heterogeneity, the I2 statistic was estimated with results ≥0.75 reflecting high heterogeneity [21]. Subgroup analyses were performed comparing manual therapy and exercises (with or without other treatments) to corticosteroid injections. Sensitivity analyses were conducted to investigate the sources of heterogeneity, examining studies with ‘low’ or ‘moderate quality’ (PEDro score<7), unexpectedly large treatment effect sizes, as well as studies presenting significant heterogeneity at baseline for participant characteristics. Statistical significance (p) was set at <0.05.

The Grading of Recommendations Assessment, Development and Evaluation (GRADE) methodology was used to assess the certainty of evidence [21]. Initially, evidence was evaluated as high certainty and was downgraded for each of the following reasons if there was (i) a high risk of bias (PEDro score <7) in most (>75%) of the eligible studies; (ii) inconsistency (substantial heterogeneity on the point estimates, statistical heterogeneity and I2 >50%); (iii) imprecision (sample does not reflect inclusion criteria of the review, CIs limit crosses the effect size of 0.5); (iv) indirectness (trials including indirect comparisons); and (v) publication bias (asymmetry in funnel plots) [16].

For function, we considered the minimal clinically important difference (MCID) with a 100-point scale, with a mean change of 10.2 points for DASH [22], 11 points for SPADI [23], and 13 points for OSS and WORC [24], respectively. For pain intensity, we defined a 30% improvement from the pooled weighted mean of the baseline [25,26]. To compute success rates in the Global Rating of Change (GROC), the responses of ‘completely recovered’ and ‘much improved’ were counted as successes [20,26].

Results

Study Selection

**Figure 1:**
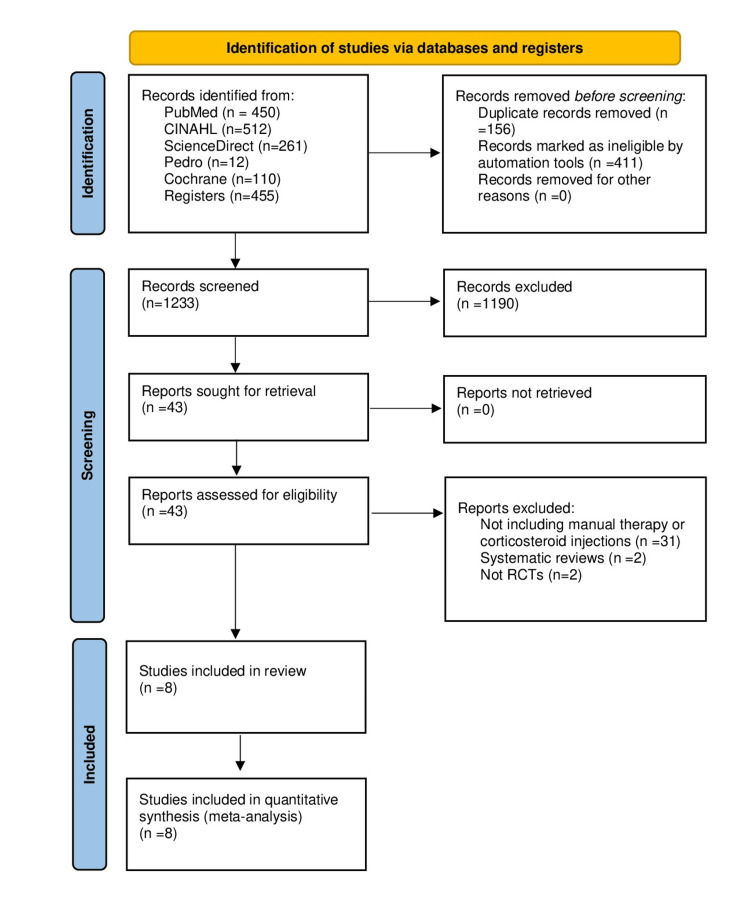
PRISMA study selection flow chart. PRISMA, preferred reporting items for systematic reviews and meta-analyses.

The search of the literature resulted in 1,233 relevant records. Screening titles and abstracts left 43 records for full-text evaluation, of which 35 studies did not meet the eligibility criteria. Eight RCTs were finally included [6,11,27-32]. The search results are shown in Figure 1. The total number of participants was 946 (mean age: 53.8 years, 52% female), and the sample size ranged from 50 to 232 participants. Based on the available demographic characteristics, the mean duration of patients’ symptoms was 5.4 months [6,30-32]. Various clinical diagnostic criteria were used to confirm the diagnosis of SAPS among the eligible studies, including shoulder pain and/or restriction during glenohumeral joint movements [6,11,27-32], positive Neer’s test or Hawkins-Kennedy test, and painful arc [6,11,31]. 

Description of the Studies

Table 1 shows the characteristics and main results of the included studies. The effectiveness of EMT compared with corticosteroid injections was evaluated in seven RCTs [6,27-32]. Two studies investigated the effectiveness of EMT against a combined approach with EMT and corticosteroid injections [11,27]. One study investigated EMT combined with corticosteroid injections against corticosteroid injections alone [27]. The total number of physiotherapy sessions ranged between 6 and 12 visits during a period of 3 to 18 weeks (Table 1). The EMT programme included glenohumeral and scapular mobilization techniques, stretching, strengthening, proprioception, or stabilization exercises for glenohumeral and scapulothoracic muscles (Table 1). Three of the included studies used a multimodal physiotherapy treatment including electrotherapy, ultrasound, ice, or hot packs in addition to the EMT programme [28,29,32]. Five of the eligible studies provided a home exercise programme (HEP) for patients following the EMT approach [7,9,11,27,32]. However, only two studies used a HEP in addition to the corticosteroid injection intervention [11,31]. Five studies used 1 to 3 injections of 40 mg methylprednisolone mixed with lidocaine [27-30,32], two studies used 1 cc triamhexal mixed with lidocaine [6,11], and one study used 20 mg triamcinolone acetonide mixed with lidocaine [31]. The injections were administered by physiotherapists [6,11], orthopaedic surgeons [6,11], general practitioners (GPs) [28,29], and a rheumatologist [32]. Only one study reported a few adverse reactions occurring after EMT, while more frequent adverse reactions were recorded with corticosteroid injections (facial flashing and irregular menstrual bleeding) [29].

**Table 1:** Included studies, demographics and results. n, sample size; EMT, exercise and manual therapy; CI, corticosteroid injection; NSAIDs, non-steroid anti-inflammatory drugs; GP, general practitioner; mg, milligram; HEP, Home Exercise Programme; OSS, Oxford Shoulder Score; SF-36, Short-Form-36; VAS, Visual Analogue Scale; DASH, Disability of the Arm Shoulder and Hand questionnaire; SPADI, Shoulder Pain and Disability Index; WORC, Western Ontario Rotator Cuff index; GROC, Global Rating of Change.

Study (year)	Total sample size (n) and age	Interventions	Length of follow-up	Outcome measures	Results
Cloke et al., (2008) [27]	112 total (54.5 y) EMT: n=27; (7 lost to follow-up) CI: n=28; (2 lost to follow-up) CI+ EMT: n=22; (5 lost to follow-up) Control: n=20; (7 lost to follow-up)	EMT: 6 sessions of a specific programme and a HEP during a maximum 18-week period. CI: A course of injections of corticosteroid (40 mg of methylprednisolone) and local anaesthetic (10 mL of 1% lidocaine) into the subacromial space for a maximum of 3 injections at 6-week intervals. Control: NSAIDs	18 weeks and 52 weeks	OSS; SF-36	No significant between-group differences in function and QoL at all follow-up times.
Crawshaw et al., (2010) [31]	232 total EMT: n=117 (57.3 ±10.3y); (11 lost to follow-up) CI: n=115 (54.9 ±10y); (19 lost to follow-up)	EMT: Physiotherapists chose from six different mobilisation techniques and 23 exercises according to the patient's needs; a HEP was provided CI: Physiotherapists performed all injections (20 mg triamcinolone acetonide mixed with 4.5 ml 1% lidocaine; could be repeated after 6 weeks; a HEP was provided	1, 6, 12 and 24 weeks	SPADI, GROC	No significant between-group differences at 12 and 24 weeks. CI group showed significantly better results at 1- and 6-weeks (p<0.05).
Daghiani et al., (2022) [6]	50 total EMT: n=25 (45.9 ±13.9y); (4 lost to follow-up) CI: n=25 (44.8 ±12.9y); (2 lost to follow-up)	EMT: glenohumeral and scapular mobilisation techniques, stretching, strengthening, proprioception and stabilisation exercises for glenohumeral and scapulothoracic muscles, sessions/week for 4 weeks CI: An orthopaedic surgeon performed all injections; unguided, landmark-based injection to inject 1cc triamhexal and 2 cc lidocaine	4, 12 and 26 weeks	VAS; Quick-DASH; SPADI; WORC; GROC	EMT group showed significantly better improvement in function and GROC at all follow-up times. No significant between-group difference in pain intensity.
Ginn and Cohen (2005) [32]	82 total EMT: n=26 (55.4y); (4 lost to follow-up) CI: n=29 (57.4y); (3 lost to follow-up) Ex.: n=27 (52.6y); (3 lost to follow-up)	EMT: twice a week for 5 weeks; electrophysical modalities, passive joint mobilisation and ROM exercises; an HEP was provided CI: An injection of 40-mg methyl prednisone acetate into the sub-acromial space under local anaesthesia with lignocaine by a rheumatologist Ex.: stretching strengthening, proprioception and stabilisation exercises for glenohumeral and scapulothoracic muscles	5 weeks	VAS ; Shoulder Function ; GROC	No significant between-group difference in pain intensity, function and treatment success.
Hay et al., (2003) [28]	207 EMT: n=103 (57.5 ±13y); (4 lost to follow-up) CI: n=104 (57.6 ±14y); (7 lost to follow-up)	EMT: 8 sessions (20 minutes) within a 6-week period; shoulder mobilisation, advice, active shoulder exercises, ultrasound, manual therapy and a HEP. CI: an injection of 40 mg of methylprednisolone mixed with 4ml 1% lidocaine (lignocaine) into the subacromial space by their GP. A second injection was offered if the symptoms did not change	6 and 26 weeks	Shoulder disability questionnaire; VAS; GROC	No significant between-group differences in function and pain intensity. EMT group showed better success rates at 26 weeks (7%).
Raeesi et al., (2022) [11]	50 total EMT: n=25 (45.2 ±13.1y); (3 lost to follow-up) CI +EMT: n=25 (50.6 ±12.6y); (4 lost to follow-up)	EMT: 12 sessions over 4 weeks; manual therapy techniques, stretching and strengthening exercises for glenohumeral and scapulothoracic muscles, dynamic and static scapular control exercises, and home exercises and a HEP. CI+EMT: an injection of of 1 cc triamhexal mixed with 2 cc lidocaine, was injected using an anterolateral approach with a 20mm needle by an orthopaedic surgeon + EMT	4, 12 and 26 weeks	VAS; DASH; SPADI; WORC; GROC	CI+ EMT group showed significantly better improvement than EMT alone in pain intensity, function and GROC at short- and mid-term.
Rhon et al., (2014) [30]	104 total EMT: n=52 (40 ±12y); (6 lost to follow-up) CI: n=52 (42 ±12y); (0 lost to follow-up)	EMT: 2 sessions per week for 3 weeks; joint and soft-tissue mobilisations; manual stretches; contract–relax techniques; and reinforcing exercises directed to the shoulder girdle or thoracic or cervical spine and HEP CI: An injection of 40 mg of triamcinolone acetonide to the subacromial space by a GP. Up to 3 injections	4, 12, 26 and 52 weeks	NRPS; SPADI; GROC	No significant between-group differences in function, pain intensity and treatment success at all follow-up times.
van der Windt et al., (1998) [29]	109 total EMT: n=56 (57.3 ±10.2y); (2 lost to follow-up) CI: n=53 (60.2 ±10.7y); (4 lost to follow-up)	EMT: 12 sessions (30 minutes); passive joint mobilisation and exercise treatment; ice or hot packs, or electrotherapy if needed CI: An injection of 40 mg of triamcinolone acetonide to the subacromial space by a GP. Up to 3 injections	3, 7, 16, 26 and 52 weeks	Shoulder disability questionnaire; VAS; GROC; ROM	CI group showed significantly better improvement than EMT alone in pain intensity, function and GROC at all follow-up times.

Risk of Bias

Based on the quality assessment, seven of the eligible trials were rated as ‘high’ and one as ‘moderate quality’ (Table 2). One study lacked a concealed allocation, two did not ensure blinding of the outcome assessors, and one trial presented substantial (>15%) losses at follow-up (Table 2). Due to the nature of the interventions, none of the studies ensured the blinding of the therapists and participants.

**Table 2:** PEDro scale items assessment with the total scores of eligible studies. PEDro, Physiotherapy Evidence Database.

	1	2	3	4	5	6	7	8	9	10	11	Total score
van der Windt et al., (1998) [29]	+	+	+	+	-	-	+	+	+	+	+	8/10
Hay et al., (2003) [28]	+	+	+	+	-	-	+	+	+	+	+	8/10
Cloke et al., (2008) [27]	+	+	+	-	-	-	-	-	+	+	+	5/10
Ginn and Cohen (2005) [32]	+	+	-	+	-	-	+	+	+	+	+	7/10
Crawshaw et al., (2010) [31]	+	+	+	+	-	-	-	+	+	+	+	7/10
Rhon et al., (2014) [30]	+	+	+	+	-	-	+	+	+	+	+	8/10
Raeesi et al., (2022) [11]	+	+	+	+	-	-	+	+	+	+	+	7/10
Daghiani et al., (2022) [6]	+	+	+	+	-	-	+	+	+	+	+	8/10
1. Eligibility criteria were specified; 2. Subjects were randomly allocated to groups (in a crossover study, subjects were randomly allocated an order in which treatments were received); 3. Allocation was concealed; 4. The groups were similar at baseline regarding the most important prognostic indicators; 5. There was blinding of all subjects; 6. There was blinding of all therapists who administered the therapy; 7. There was blinding of all assessors who measured at least one key outcome; 8. Measures of at least one key outcome were obtained from more than 85% of the subjects initially allocated to groups; 9. All subjects for whom outcome measures were available received the treatment or control condition as allocated or, where this was not the case, data for at least one key outcome was analysed by “intention to treat”; 10. the results of between-group statistical comparisons are reported for at least one key outcome; 11. the study provides both point measures and measures of variability for at least one key outcome. Note: The first item relates to external validity and the remaining 10 items are used to calculate the total score, which ranges from 0 to 10 + Yes - No.												

Main Results of Meta-Analyses

**Figure 2:**
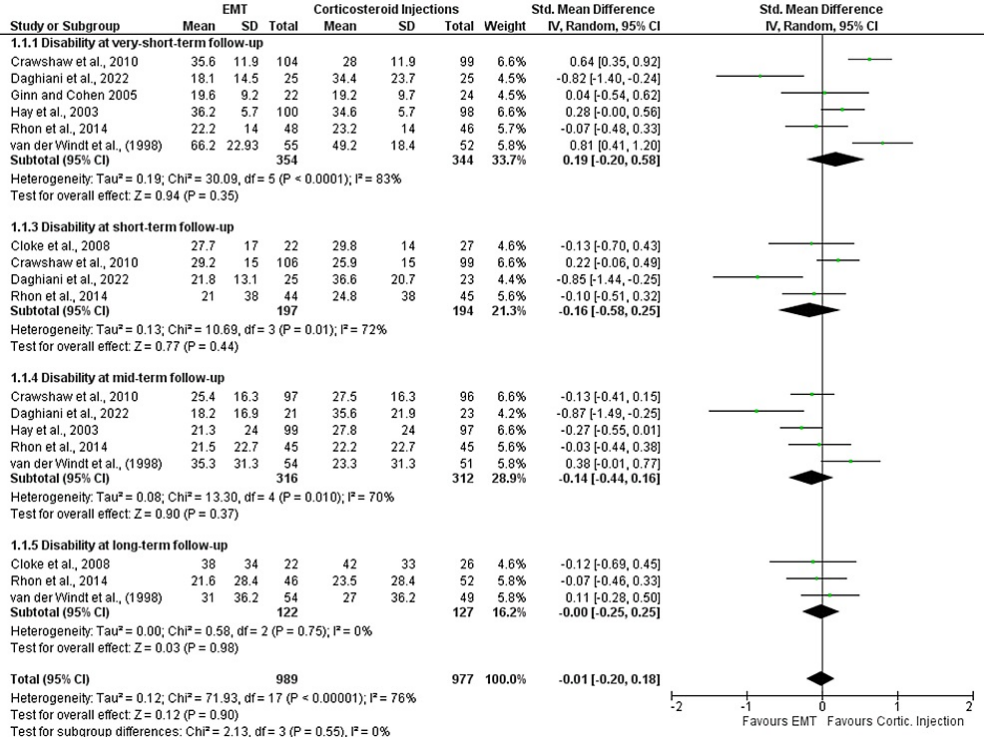
Forest plots for the effectiveness of EMT compared with corticosteroid injections in shoulder disability in patients with SAPS. IV, inverse variance; CI, confidence intervals; EMT, exercise and manual therapy, SAPS, subacromial pain syndrome; Cortic., corticosteroid.

**Figure 3:**
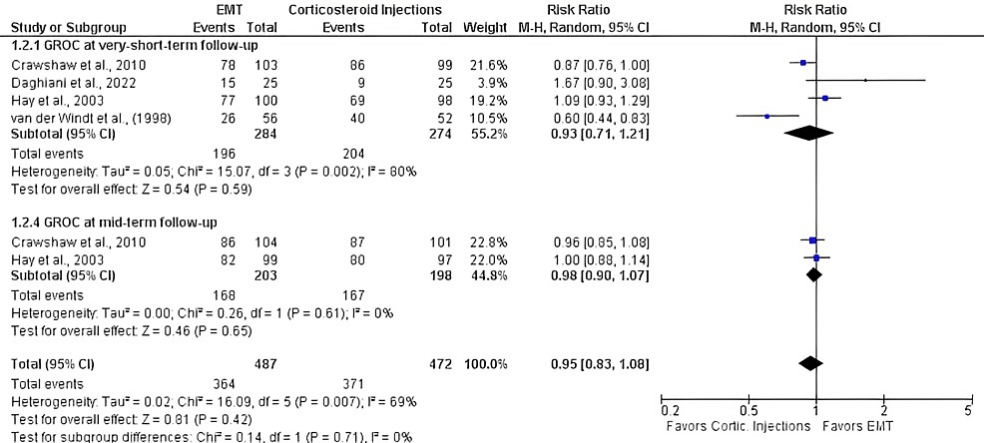
Forest plots for the effectiveness of EMT compared with corticosteroid injections in GROC in patients with SAPS. IV, inverse variance; CI, confidence intervals; EMT, exercise and manual therapy, GROC, Global Rating of Change; SAPS, subacromial pain syndrome; Cortic., corticosteroid.

**Figure 4:**
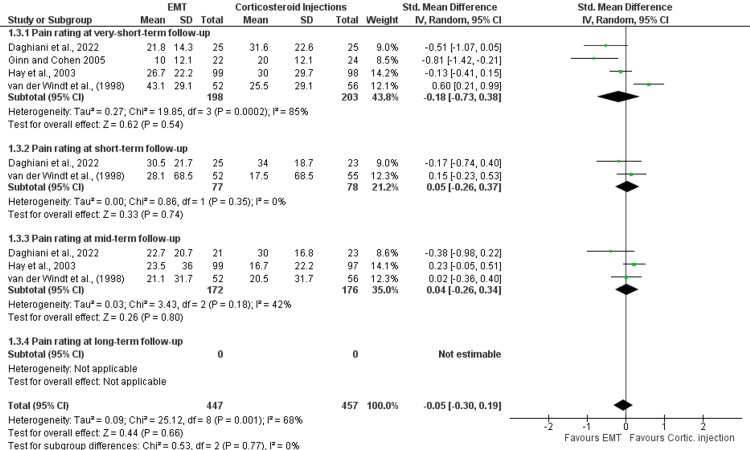
Forest plots for the effectiveness of EMT compared with corticosteroid injections in pain rating in patients with SAPS. IV, inverse variance; CI, confidence intervals; EMT, exercise and manual therapy, SAPS, subacromial pain syndrome; Cortic., corticosteroid.

Seven studies that were included in the quantitative synthesis made direct or indirect comparisons between EMT and corticosteroid injections [6,27-32]. Follow-up occasions fluctuated between 1 week and 1 year, and the mean age of the patients (n = 812) was 54.11 years. All the eligible studies evaluated shoulder disability using a variety of outcome measures (i.e., SPADI, DASH, quick-DASH, OSS, or a shoulder disability questionnaire). Based on the meta-analysis results, there was no difference between EMT and corticosteroid injections in disability at very short- (SMD: 0.19; 95%CI: -0.20, 0.58), short- (SMD: -0.16; 95%CI: -0.58, 0.25), mid- (SMD: -0.14; 95%CI: -0.44, 0.16) and long-term (SMD: 0.00; 95%CI: -0.25, 0.25) follow-up (Table 3). Also, four of the eligible trials assessed self-perceived recovery using a GROC scale, reporting no significant differences between the comparators at very short- (risk ratio {RR}: 0.93; 95%CI: 0.71, 1.21) and mid-term (RR: 0.98; 95%CI: 0.90, 1.07) follow-up (Table 3) [6,28,29,31]. Similarly, four studies evaluated pain intensity using either a VAS or NRPS, suggesting no difference between EMT and corticosteroid injections at very short- (SMD: -0.18; 95%CI: -0.73, 0.38), short- (SMD: 0.05 95%CI: -0.26, 0.37), and long-term (SMD: 0.04 95%CI: -0.26, 0.34) follow-ups [6,28,29,32]. Forest plots for the effectiveness of EMT compared with corticosteroid injections in shoulder disability, self-perceived recovery, and pain intensity are shown in Figures 2, 3 and 4. All outcomes were rated from very low to moderate certainty of evidence.

**Table 3:** Self-perceived improvement, disability, pain intensity for exercise and manual therapy compared with corticosteroid injections in patients with subacromial shoulder pain. ^1^ Inconsistency between included studies due to increased statistical heterogeneity; ^2 ^Indirectness of interventions among the included studies; ^3^ Imprecision results of the included studies. CI, confidence interval; MD, mean difference; SMD, standardised mean difference; EMT, exercise and manual therapy; CI, corticosteroid Injection; GROC, Global Rating of Change; RR, risk ratio.

Outcomes	No of studies	Comparisons		Effects estimate (95%CI); P value	Certainty (GRADE)	Heterogeneity
		Average estimate / assumed risk in EMT group	Average estimate / assumed risk in CI group			
GROC (Very short-term follow-up)	4	196/284 (69%) participants reported satisfactory recovery	204/274 (74.4%) participants reported satisfactory recovery	RR: 0.93 (0.71, 1.21); P=0.69	⊕^1,2,3^ Very Low	Chi²:15.7 (P=0.002); I² = 80%
GROC (Mid-term follow-up)	2	168/203 (82.7%) participants reported satisfactory recovery	167/198 (84.3%) participants reported satisfactory recovery	RR: 0.98 (0.90, 1.07); P=0.65	⊕⊕⊕^2^ Moderate	Chi²:0.26 (P=0.61); I² = 80%
Disability (Very short-term follow-up)	6	Mean disability score was 35.9 (range 18.1 to 66.2) in 354 participants	Mean disability score was 32.2 (range 19.2 to 49.2) in 344 participants	SMD: 0.19 (-0.20, 0.58); P=0.35	⊕^1,2,3^ Very Low	Chi²:30.9 (P<0.001); I² = 83%
Disability (Short-term follow-up)	4	Mean disability score was 26.2 (range 19 to 29.2) in 197 participants	Mean disability score was 27.4 (range 24.8 to 36.6) in 194 participants	SMD: -0.16 (-0.58, 0.25); P= 0.44	⊕^1,2,3^ Very Low	Chi²:10.29 (P=0.01); I² =72%
Disability (Mid-term follow-up)	5	Mean disability score was 24.7 (range 18.2 to 35.3) in 316 participants	Mean disability score was 28 (range 16.3 to 31.3) in 312 participants	SMD: -0.14 (-0.44, 0.16); P=0.37	⊕^1,2,3^ Very Low	Chi²:10. 9 (P=0.01); I² =70%
Mean change in pain rating (Mid-term follow-up)	3	Mean disability score was 28.7 (range 21.3 to 35.3) in 122 participants	Mean disability score was 28.6 (range 22.2 to 35.6) in 127 participants	SMD: -0.00 (-0.25, 0.25); P=0.98	⊕^1,2,3^ Very Low	Chi²:0.58 (P=0.75); I² =0%
Mean change in pain rating (Very short-term follow-up)	4	Mean pain score was 28.5 (range 10 to 43.1) in 198 participants	Mean pain score was 27.7 (range 20 to 25.5) in 203 participants	SMD: -0.18 (-0.73, 0.38); P=0.54	⊕⊕^2,3^ Low	Chi²:19.85 (P<0.001); I² = 85%
Mean change in pain rating (Short-term follow-up)	2	Mean pain score was 28.8 (range 28.1 to 30.5) in 77 participants	Mean pain score was 22.3 (range 17.5 to 34) in 78 participants	SMD: 0.05 (-0.26, 0.37); P=0.74	⊕⊕^2,3^ Low	Chi²: 0.86, (P = 0.35); I² = 0%
Mean change in pain rating (Mid-term follow-up)	3	Mean pain score was 22.7 (range 21.1 to 23.5) in 172 participants	Mean pain score was 19.6 (range 16.7 to 30) in 176 participants	SMD: 0.04 (-0.26, 0.34); P=0.80	⊕⊕^2,3^ Low	Chi²: 3.43 (P = 0.18); I² = 42%

**Figure 5:**
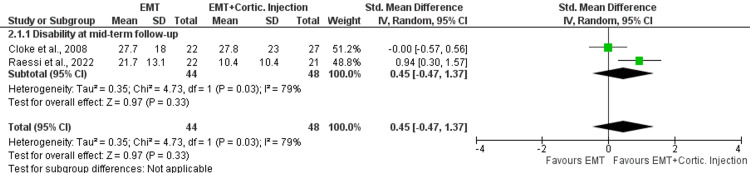
Forest plots for the effectiveness of the addition of corticosteroid injections to EMT compared with corticosteroid injections alone in shoulder disability in patients with SAPS. IV, inverse variance; CI, confidence intervals; EMT, exercise and manual therapy, SAPS, subacromial pain syndrome; Cortic., corticosteroid.

Two trials performed a direct comparison between EMT combined with corticosteroid injections and EMT alone [11,27]. The mean age of the participants analysed (n = 92) was 51.5 years. Quantitative synthesis was possible only for disability scores at mid-term follow-up, suggesting no differences between the interventions (SMD: 0.45; 95%CI: -0.47, 1.37) based on the low quality of evidence (Figure 5). One study evaluated an EMT programme combined with corticosteroid injections against corticosteroid injections alone reporting no significant differences in improving function at mid-term follow-up (MD: 2.70; 95%CI: -7.70, 13.10).

We performed a sensitivity analysis excluding studies either using a multimodal treatment approach or with a ‘moderate quality’ PEDro score, which resulted in no change in the effect estimate of outcomes.

Discussion

We analysed eight RCTs with 946 participants (mean age 53.8 years), and all these studies showed ‘high’ (7 trials) or ‘moderate’ (1 trial) methodological quality. Due to the increased statistical heterogeneity, imprecision, and indirectness of interventions, the GRADE of evidence ranged from very low to moderate quality. The most important finding of our review is that a treatment approach based on EMT has similar effects in reducing pain and improving function and self-perceived recovery compared with corticosteroid injections in patients with SAPS at all follow-up times (very low to moderate quality of evidence). In addition, the combination of EMT and corticosteroid injections does not provide better results in reducing shoulder disability compared to corticosteroid injections or EMT alone at mid-term follow-up (low quality of evidence).

Multiple systematic reviews and meta-analyses have evaluated the effectiveness of each approach in the management of patients with SAPS [10,13,33-35]. Based on moderate and high levels of evidence, the addition of manual therapy to exercises is considered an effective intervention in reducing pain and disability, at least in the short term [2,14]; however, the effect sizes for the EMT compared with control conditions are small to moderate [14]. Similar results have been reported for the clinical significance of corticosteroid injections as a stand-alone treatment or in addition to an EMT treatment regime [36]. To our knowledge, only two reviews have investigated the comparable effects of both approaches in patients with SAPS. In agreement with our findings, a previous Cochrane review suggested that EMT and corticosteroid injections may produce similar effects on pain and function; nevertheless, a quantitative synthesis was lacking [8]. At the same time, another meta-analysis differed from our conclusions, supporting significant short-term benefits in favour of corticosteroid injections compared to EMT in shoulder function [37]. However, their results should be interpreted with caution due to the limited number of studies analysed (3 trials) compared to the present quantitative synthesis (8 trials).

Although our findings suggest no difference between EMT and corticosteroid injections in all outcomes and follow-up occasions, the certainty of the evidence was downgraded, mainly due to substantial clinical and statistical heterogeneity [21]. Notably, the manual therapy approach varied widely among the eligible studies in terms of the type (glenohumeral and scapular mobilisations, specific soft tissue mobilisation, passive shoulder mobilisation, manual stretching, etc.), number of sessions (6 to 12), and duration of treatment(4 to 18 weeks) [6,11,27-32]. Similarly, a wide range of exercises was used among the eligible trials including strengthening, stabilization, stretching and proprioception training programmes. However, the optimal dose, i.e. the total number of repetitions, sets, number of exercises, frequency, intensity, and duration was not clearly described [6,11,27-32]. In the same vein, increased clinical heterogeneity was noted in the healthcare professionals providing the corticosteroid injections (physiotherapists, GPs, orthopaedic consultants, and rheumatologists) and the type of medication used as well (Table 1).

The substantial inconsistency observed in the quantitative synthesis may also be attributed to the patient characteristics that were included among the eligible studies. For example, most trials either did not report critical demographic characteristics of participants (such as type or duration of symptoms) [27,28,31], or their sample varied greatly in terms of chronicity [11,29]. Evidence suggests that symptom duration and severity of pain are critical prognostic indicators for treatment success in patients with shoulder pain [31,38]. Additionally, several other factors might have influenced the effectiveness of the comparable interventions, including the wide variety of underlying structures involved in the pathogenesis of the condition [27], patients’ psychological status, and treatment expectations [39,40].

The clinical implications of our study suggest that an EMT programme with or without other physical therapy modalities can produce similar effects in function and pain intensity to corticosteroid injections in the management of patients with SAPS. Based on limited evidence, if early pain relief is a priority, providing a corticosteroid injection on top of EMT might offer significantly greater improvements at the one-week follow-up; however, pain intensity and function disability changes are similar between the comparable interventions at very short-, short-, mid-, and long-term follow-up times. Furthermore, the addition of corticosteroid injections to EMT does not seem to alter the clinical benefits compared with EMT or corticosteroid injections alone. Considering that EMT presents fewer adverse reactions, cost-effectiveness and other associated health benefits, it is suggested to be prioritised in the management of patients with SAPS [2,14].

Limitations and Future Research

Our study findings should be interpreted considering some limitations. First, the increased clinical heterogeneity in specific interventions, healthcare practitioners, and patients’ characteristics (symptom duration) among the studies may be a potential confounder factor of the outcomes [21]. Although we analytically recorded all these parameters, a subgroup analysis for these factors was not feasible. Second, the limited number of trials did not permit further evaluation of publication bias by generating funnel plots. Third, the comparison of EMT alone or in combination with a multimodal physiotherapy programme possibly influenced precise estimations.

Future studies should clearly describe all aspects of interventions used, such as the type of manual therapy techniques and loading interventions, the number of exercises, and the criteria of treatment progression. Further investigation of the effectiveness of the two comparators in subgroups of patients with SAPS based on baseline disability or symptom duration seems necessary.

## Conclusions

Based on very low to moderate certainty of evidence, the addition of manual therapy to exercise interventions is equally effective compared with corticosteroid injections in improving all outcomes in patients with SAPS at all follow-up periods. Based on low certainty of evidence, the combination of both interventions compared with EMT or corticosteroid injections alone has similar effects on improving function at the mid-term follow-up. The limited research details and heterogeneity of the EMT components did not allow us to provide clinical recommendations for the optimal EMT treatment approach. Until further research confirms which patients can benefit more from each intervention, clinicians can equally recommend an EMT, corticosteroid injection, or both in the management of patients with SAPS.

## Appendices

**Table 4:** Search strategy

Databases: MEDLINE, PubMed, CINAHL, EMBASE, PEDro, ScienceDirect, Cochrane Library	
1	Shoulder impingement syndrome
2	Shoulder pain
3	Subacromial pain syndrome
4	Rotator cuff tendinopathy
5	Supraspinatus or supra-spinatus tendinopathy
6	Shoulder bursitis
7	Shoulder tendinitis
8	1 or 2 or 3 or 4 or 5 or 6 or 7
9	Physiotherapy
10	Physical therapy
11	Rehabilitation
12	Exercise
13	Resistance training
14	Training
15	Exercise therapy
16	Manual therapy
17	Mobilisation
18	Manipulation
19	Massage
20	9 or 10 or 11 or 12 or 13 or 14 or 15 or 16 or 17 or 18 or 19
21	Cortisone
22	Corticosteroid injection
23	Glucocorticoids
24	Injection
25	21 or 22 or 23 or 24
26	8 and 20 and 25
